# Monocyte to large platelet ratio as a diagnostic marker for acute pulmonary embolism

**DOI:** 10.1016/j.ahjo.2026.100951

**Published:** 2026-10-13

**Authors:** Mohamed K.A. Awad, Reem H. El Kabarity, Hadeel Magdy Abdelhamid, Azza M. Youssef

**Affiliations:** a Division of Critical Care, Department of Anesthesia and Critical Care, Faculty of Medicine, Ain Shams University, Cairo, Egypt; b Department of Anesthesia and Critical Care, Faculty of Medicine, Ain Shams University, Cairo, Egypt

**Keywords:** Pulmonary embolism, MLPR (Monocyte-to-Lymphocyte Platelet Ratio), NLR, Hematologic biomarkers

## Abstract

**Background:**

Pulmonary embolism (PE) is a potentially life-threatening diagnosis that commonly presents to the emergency department with nonspecific symptoms. Despite advances in diagnostic algorithms, rapid identification remains challenging, particularly when definitive imaging is delayed or unavailable. Simple and readily available biomarkers may assist in the early evaluation of patients with suspected PE. This study investigated the diagnostic value of the monocyte-to-large platelet ratio (MLPR), a novel inflammation- and thrombosis-related biomarker, in acute PE.

**Methods:**

This retrospective exploratory study reviewed 115 medical records of patients presenting with suspected PE to the emergency department between January and December 2024 who underwent computed tomography pulmonary angiography. After applying exclusion criteria, 53 patients were included (25 PE, 28 non-PE). Admission complete blood count parameters, including MLPR, monocyte and lymphocyte counts, and derived ratios, were analyzed.

**Results:**

Age and sex distribution were similar between groups. Total leukocyte, monocyte, neutrophil, and lymphocyte counts were significantly higher in the PE group, alongside significantly elevated MLPR (1.19 ± 0.45 vs. 0.61 ± 0.39; *P* < 0.001), while NLR did not differ significantly (*P* = 0.4235). MLPR demonstrated the highest diagnostic performance among individual hematological markers (AUC 0.854, 95% CI 0.752–0.956; *P* < 0.001). Combining MLPR with lymphocyte count further improved diagnostic accuracy (AUC 0.907, 95% CI 0.827–0.986), achieving 92% sensitivity and 85.7% specificity.

**Conclusion:**

MLPR is a promising, readily available biomarker for diagnosing acute PE, with enhanced performance when combined with lymphocyte count.

## Highlights

• ◉ **MLPR is significantly** **higher** in acute PE patients compared to non-PE patients (1.19 ± 0.45 vs. 0.61 ± 0.39; *P* < 0.001).
• ↗ **MLPR outperforms** other individual hematologic markers in diagnosing acute PE (AUC 0.854, 95% CI 0.752–0.956; P < 0.001).
• ♟ Combining **MLPR with lymphocyte count** further improves diagnostic accuracy (AUC 0.907, 95% CI 0.827–0.986; P < 0.001).
• ✓ **Bootstrap validation** confirmed the stability of the combined MLPR–lymphocyte model (AUC 0.907, 95% CI 0.826–0.988).
• ☷ MLPR is a **simple**, **inexpensive**, **and readily available** biomarker that may aid the early PE diagnosis in resource-limited settings.

## 1 Introduction

Pulmonary embolism (PE) arises when a thrombus, typically originating from another part of the body, obstructs blood flow in the pulmonary artery or its branches. Most commonly, this thrombus forms in the deep veins of the lower limbs, a condition known as deep vein thrombosis (DVT). PE usually results from a fragment of this thrombus detaching and traveling to the pulmonary circulation. Although rare, PE can also be caused by embolization of other substances such as air, fat, or tumor cells. Together, PE and DVT constitute the clinical spectrum of venous thromboembolism (VTE) [1].

Since the majority of PEs originate from DVTs, the risk factors for PE mirror those of DVT. These risk factors can be understood through Virchow's triad, which includes hypercoagulability, venous stasis, and endothelial injury [2]. The annual incidence of PE ranges between 39 and 115 cases per 100,000 individuals, while DVT incidence varies from 53 to 162 per 100,000. Acute PE ranks as the third most common cardiovascular condition after coronary artery disease and stroke [3], [4].

Delayed diagnosis of pulmonary embolism is associated with worse outcomes, driven by factors such as treatment delays and patient-related variables, including advanced age. The nonspecific nature of PE symptoms and their overlap with comorbid conditions further impede timely recognition. In developing countries, limited access to diagnostic tools is a major factor in late diagnosis, highlighting the need for rapid, simple, and reliable laboratory tests to facilitate earlier treatment [5].

Because no single clinical feature or test definitively diagnoses PE, physicians must integrate clinical presentation with biochemical and imaging findings [6]. Imaging techniques therefore play complementary roles in diagnosis. Computed tomography pulmonary angiography is highly effective for confirming PE, whereas ventilation–perfusion lung scanning may be used for both confirmation and exclusion but frequently yields indeterminate results, particularly in patients with underlying lung disease [7], [8]. Magnetic resonance angiography has also been evaluated for PE diagnosis but is not recommended as a first-line modality because of limited sensitivity, restricted availability in emergency settings, and a high rate of inconclusive examinations [9]. Accordingly, the selection of imaging tests is guided by clinical probability and patient-specific factors that influence diagnostic performance [7].

Laboratory investigations serve as adjuncts to imaging in the diagnostic workup of PE. D-dimer which is useful in excluding PE in patients with low clinical probability is widely used but has limited specificity, which reduces its diagnostic accuracy [10], [11], [12]. Other biomarkers like brain natriuretic peptide (BNP), and troponin have been studied for their diagnostic and prognostic roles in PE, but none serve as a standalone diagnostic tool [13], [14], [15], [16]. Consequently, platelet- and hematology-based indices, reflecting systemic inflammation, have emerged as promising candidate markers, such as the neutrophil-to-lymphocyte ratio (NLR) and monocyte-related measures [17], [18], [19], [20]. Among these markers, the monocyte-to-large platelet ratio (MLPR) is of particular interest. Which is a composite hematological index that relates circulating monocyte abundance to the presence of large platelets. Monocytes may contribute to thrombo-inflammation through interactions with platelets and endothelial cells and through the release of proinflammatory and procoagulant mediators [20]. Large platelets are generally considered more reactive and may have greater prothrombotic potential because of their increased content of platelet granules and activation-related mediators [21], [22]. The interaction between inflammation, platelet activation, and coagulation is relevant to the pathophysiology of acute PE; therefore, MLPR may reflect aspects of the inflammatory and thrombotic response associated with PE, although the biological mechanisms underlying this association remain to be established.

This exploratory study primarily aimed to evaluate the diagnostic performance of MLPR for identifying acute PE in patients presenting with suspected PE. Secondary aims were to compare MLPR with other hematological indices and to explore its potential incremental diagnostic value when combined with clinical pretest probability using the Wells score. We hypothesized that MLPR would demonstrate discriminatory ability for acute PE and that its combination with complementary laboratory or clinical parameters could provide additional diagnostic information.

## 2 Methodology

### 2.1 Study design and setting

This retrospective, exploratory cohort study was conducted to assess the diagnostic value of the Monocyte-to-Large Platelet Ratio (MLPR) as a biomarker for pulmonary embolism (PE). Data were collected from the emergency department of Ain Shams University Hospital between January 2024 and December 2024.

### 2.2 Participants

Patients were eligible for inclusion if they presented to the emergency department with suspected PE according to wells' Criteria (Patients with a low clinical probability underwent D-dimer testing as part of the diagnostic pathway, whereas patients with a high clinical probability proceeded directly to CTPA. All included patients underwent computed tomography pulmonary angiography (CTPA), which was used as the reference standard for confirming or excluding PE), had a complete blood count (CBC) with white blood cell differentiation obtained at initial presentation and prior to initiating treatment, and underwent confirmatory computed tomography pulmonary angiography (CTPA) as part of standard diagnostic evaluation. Only samples processed on the analyzer capable of reporting large platelet parameters were analyzed.

### 2.3 Exclusion criteria

As an exploratory study, we aimed to minimize potential confounding factors that could affect hematological parameters. Therefore, patients were excluded from the study if they had a known history of hematological disorders such as myeloproliferative diseases, myelofibrosis, Glanzmann thrombasthenia, May–Hegglin anomaly, or Bernard–Soulier syndrome, or clinical suspicion of disseminated intravascular coagulation (DIC). May–Hegglin anomaly was excluded when a documented prior diagnosis was present because of its association with abnormally large platelets and thrombocytopenia, which could affect MLPR calculation. Clinical suspicion of DIC was based on documented bleeding and/or laboratory evidence of consumptive coagulopathy, including thrombocytopenia and abnormal coagulation parameters. Additional exclusion criteria included prior therapeutic anticoagulation or receipt of blood transfusion within the past two months, diagnosis of acute coronary syndrome or pulmonary edema established before CTPA, active malignancy, current use of immunosuppressive therapy or oral contraceptive pills or dual antiplatelet, documented cirrhosis or decompensated advanced chronic liver disease, or advanced chronic kidney disease (eGFR <30 mL/min/1.73 m^2^) or dialysis dependence, and age < 18 years. Samples processed using laboratory analyzers that did not provide the platelet parameters required for MLPR calculation were also excluded.

### 2.4 Group classification

Based on the results of computed tomography pulmonary angiography (CTPA), patients were classified into two distinct groups. Group A included patients with a confirmed diagnosis of pulmonary embolism, while Group B consisting of patients in whom PE was excluded. This classification served as the foundation for subsequent comparative analyses of clinical and hematological parameters between the two cohorts. Deep vein thrombosis (DVT) was identified based on the available clinical documentation and lower-extremity venous duplex/compression ultrasonography when performed. The presence of DVT was assessed separately from the CTPA-based classification of PE; therefore, patients in the non-PE group could have DVT despite the absence of PE on CTPA.

### 2.5 Sample size calculation

The required sample size was determined using PASS 11 software, referencing the study by Bialas et al. [20], which reported an AUC of 0.95 for MLPR in diagnosing PE. Assuming a 25% prevalence of PE among suspected cases, a minimum of 20 patients was needed to achieve 80% power at a significance level of 0.05.

### 2.6 Ethical approval and consent to participate

This study was approved by the Research Ethics Committee (REC) FWA00017585, Faculty of Medicine, Ain Shams University, and was registered on ClinicalTrials.gov (Identifier: NCT07023705). Due to the retrospective nature of the study, the requirement for informed consent was waived. Patient medical records were reviewed to identify eligible cases. All methods were carried out in accordance with relevant guidelines and regulations, including the Declaration of Helsinki.

### 2.7 Data collection

Only patients who had undergone CTPA and pre-treatment venous blood sampling were included. The primary outcome measure was the Monocyte-to-Large Platelet Ratio (MLPR) calculated as MLPR = (monocyte count / LPC) × 100, where LPC = (PLT × PLCR) / 100. Secondary parameters included mean platelet volume (MPV), platelet distribution width (PDW), platelet-to-lymphocyte ratio (PLR), and D-dimer levels (where available).

### 2.8 Statistical analysis

No interventions were performed in this study. All analyses were conducted using STATA 18. Quantitative variables were summarized as mean ± standard deviation (SD) and median (interquartile range [IQR]). Given the limited sample size and potential non-normal distribution, continuous variables were compared using the Mann–Whitney *U* test. Categorical variables were reported as frequencies and percentages and compared using Fisher's exact test, as appropriate.

The diagnostic performance of the monocyte-to-large platelet ratio (MLPR) and other hematological markers was assessed using receiver operating characteristic (ROC) curve analysis, with calculation of the area under the curve (AUC) and corresponding 95% confidence intervals (CI). Sensitivity, specificity, positive predictive value (PPV), negative predictive value (NPV), and likelihood ratios (LR+ and LR−) were also determined.

The Wells score was calculated from the available clinical information for all included patients and was evaluated for its diagnostic performance both independently and in combination with MLPR. ROC curves were used to compare the discriminatory performance of the Wells score, MLPR, and the combined Wells score–MLPR model.

Univariable logistic regression analyses were performed to evaluate associations between candidate predictors and pulmonary embolism. Multivariable logistic regression was performed to assess the association between MLPR and PE after adjustment for Wells score, age, and gender. Given the limited number of PE events, the multivariable model was restricted to these clinically relevant variables to minimize the risk of overfitting. Multicollinearity among variables included in the multivariable model was assessed using variance inflation factors (VIFs) and tolerance values. Differences between ROC AUCs were assessed using paired ROC curve comparison.

Internal validation was performed using bootstrap resampling with 1000 iterations to assess the stability of ROC-derived diagnostic performance measures, including AUC, sensitivity, specificity, and their corresponding 95% confidence intervals.

## 3 Results

Our study initially reviewed the records of 115 patients. After applying inclusion criteria, a total of 53 patients were enrolled, of whom 25 (47%) were diagnosed with pulmonary embolism (PE), while 28 (53%) did not have PE. A total of 62 patients were excluded: 23 had missing laboratory results including platelet parameters, 9 had active malignancy receiving treatment, 8 were on dual antiplatelet therapy, 18 had active cardiac conditions, and 4 were receiving immunosuppressive therapy (Fig. 1).

**Fig. 1:**
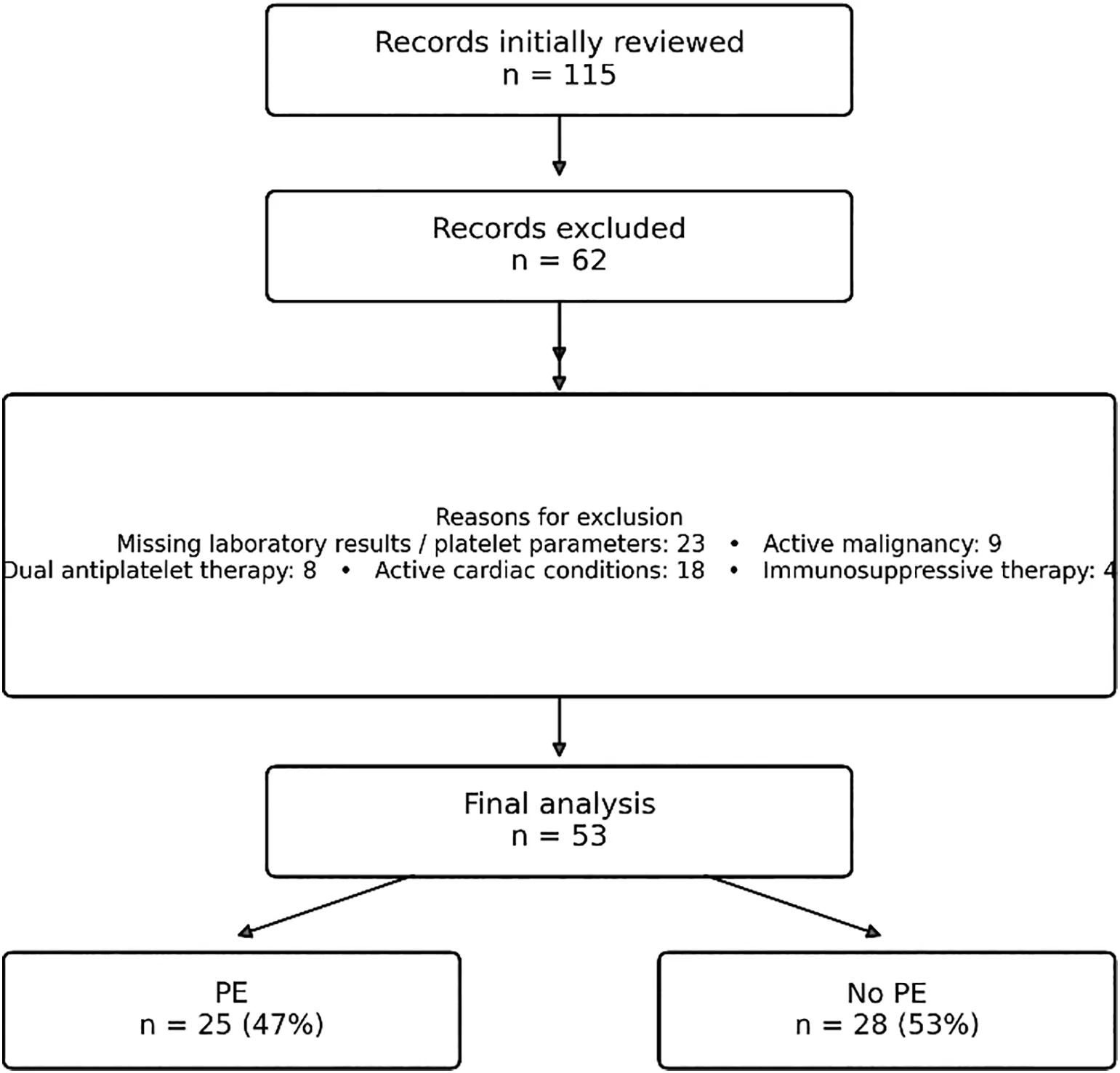
Flow diagram of patient selection.

### 3.1 Baseline characteristics

The mean age was similar between the PE group (47.4 + 16.8 years) and the non-PE group (51.5 ± 18.55 years) (*P* = 0.345). The proportion of males was 52% in the PE group and 28.5% in the non-PE group (*P* = 0.099). No statistically significant differences were found between the two groups in terms of history of deep vein thrombosis (28% vs. 21.4%, *P* = 0.230), hypertension (20% vs. 21.4%, *P* = 1.000), diabetes mellitus (12% vs. 21.4%, *P* = 0.474), history of malignancy (0% vs. 10.7%, *P* = 0.238), trauma (8% vs. 3.5%, *P* = 0.597), or recent surgery (12% vs. 3.5%, *P* = 0.333) (Table 1).

**Table 1:** Baseline characteristics of PE and non-PE groups.

Parameter	PE (25)	No PE (28)	*P* value (Fisher's or Mann–Whitney)
Age	47.4 ± 16.8 45 (33–65)	51.5 ± 18.55 51.5 (38.5–70)	0.345
Male N(%)	13 (52%)	8 (28.5%)	0.099
DVT	7 (28%)	6 (21.4%)	0.230
HTN	5 (20%)	6 (21.4%)	1.000
DM	3 (12%)	6 (21.4%)	0.474
Hx Malignancy	0 (0%)	3 (10.7%)	0.238
Trauma	2 (8%)	1 (3.5%)	0.597
Surgery	3 (12%)	1 (3.5%)	0.333

Among the 28 patients in whom PE was excluded by CTPA, the most common final diagnoses were pneumonia (9/28, 32.1%), COPD exacerbation (6/28, 21.4%), pleural effusion (4/28, 14.3%), IPF exacerbation (3/28, 10.7%), pericarditis (3/28, 10.7%), atelectasis (2/28, 7.1%), and pneumothorax (1/28, 3.6%).

Linear regression analyses exploring the relationship between various clinical factors (age, gender, DVT, hypertension, diabetes, malignancy, trauma, and surgery) and the Monocyte-to-Large Platelet Ratio (MLPR) showed no significant associations for most variables. None of the factors—age (*P* = 0.446, R^2^ = 0.011), DVT (*P* = 0.466, R^2^ = 0.010), hypertension (*P* = 0.565, R^2^ = 0.007), diabetes (*P* = 0.310, R^2^ = 0.020), trauma (*P* = 0.918, R^2^ = 0.0002), or surgery (*P* = 0.789, R^2^ = 0.001)—demonstrated significant relationships with MLPR levels, with all *p*-values exceeding 0.05. However, gender (*P* = 0.013, R^2^ = 0.116) and history of malignancy (*P* = 0.016, R^2^ = 0.109) showed statistically significant associations with MLPR levels. The low R^2^ values suggest that these clinical variables contributed minimally to MLPR variation and are unlikely to represent major confounders in its potential diagnostic role for pulmonary embolism.

### 3.2 Hematological parameters

Hematological parameters showed several significant differences between the pulmonary embolism (PE) and non-PE groups. Total leukocyte count was significantly higher in the PE group (*P* = 0.0034), along with increased monocyte (*P* < 0.001), neutrophil (*P* = 0.0296), and lymphocyte counts (*P* = 0.0004). The monocyte-to-large platelet ratio (MLPR) was also markedly elevated in PE patients (1.19 ± 0.45 vs. 0.61 ± 0.39, *P* < 0.001). The Wells score was also significantly higher in patients with PE than in those without PE (4.82 ± 1.89 vs. 3.68 ± 1.67, respectively; *P* = 0.014).

In contrast, platelet-derived indices including platelet-to-lymphocyte ratio (PLR), large platelet-to-lymphocyte ratio (LPLR), and large cell platelet count (LPC) were significantly lower in the PE group (*P* = 0.0007, *P* < 0.001, and *P* = 0.0340, respectively). Plateletcrit (PCT) showed a non-significant trend toward lower values in PE patients (*P* = 0.0855). Conventional platelet parameters, including platelet count, mean platelet volume (MPV), platelet distribution width (PDW), and platelet-large cell ratio (P-LCR), did not differ significantly between groups (*P* > 0.05). Neutrophil-to-lymphocyte ratio (NLR) was also comparable between groups (*P* = 0.4235). D-dimer levels showed no significant difference between the two groups (4.47 ± 1.98 vs. 3.05 ± 2.10 μg/mL FEU, *P* = 0.0729). Given the limited number of patients with available D-dimer measurements, this result should be interpreted cautiously and does not permit a direct comparison of D-dimer with MLPR (Table 2).

**Table 2:** Hematologic and inflammatory markers in PE vs. non-PE groups.

Parameter	PE (25)	No PE (28)	*P* value Mann Whitney
Platelets (10^3^/μL)	249.88 ± 79.97 251 (183–286)	294.57 ± 121.48 290 (209–333)	0.1438
P-LCR (%)	28.40 ± 8.21 27.1 (23.6–30.2)	30.50 ± 7.27 31.65 (24.85–34.75)	0.1318
LPC (10^3^/μL)	69.21 ± 24.16 70.48 (47.50–83.01)	84.95 ± 25.11 80.66 (68.08–105.04)	0.034*
PDW (fL)	11.89 ± 2.11 11.7 (10.3–12.7)	12.33 ± 2.06 12.5 (10.6–14.4)	0.3508
MPV (fL)	10.45 ± 1.07 10.1 (9.9–10.8)	10.67 ± 0.92 10.9 (9.8–11.2)	0.2302
PCT (%)	0.259 ± 0.078 0.27 (0.19–0.30)	0.306 ± 0.108 0.30 (0.22–0.36)	0.0855*
TLC (10^3^/μL)	10.84 ± 2.44 10.21 (9.28–12.43)	8.06 ± 3.63 6.2 (5.02–11.45)	0.0034*
Monocytes (10^3^/μL)	0.78 ± 0.26 0.78 (0.61–0.95)	0.47 ± 0.24 0.42 (0.30–0.60)	<0.001*
Neutrophils (10^3^/μL)	7.67 ± 2.16 6.88 (6.29–8.75)	6.21 ± 3.46 5.01 (3.02–8.53)	0.0296*
Lymphocytes (10^3^/μL)	2.23 ± 1.10 2.02 (1.55–2.92)	1.31 ± 0.65 1.33 (0.77–1.87)	0.0004*
MLPR	1.19 ± 0.45 1.15 (0.85–1.35)	0.61 ± 0.39 0.50 (0.35–0.87)	<0.001*
NLR	11.09 ± 25.30 3.60 (2.44–5.46)	9.99 ± 14.44 5.02 (2.33–11.25)	0.4235
PLR	118.05 ± 61.78 116.13 (84.23–159.53)	264.79 ± 222.23 181.68 (148.87–306.06)	0.0007*
LPLR	34.54 ± 15.21 31.89 (24.81–40.95)	90.90 ± 68.20 58.08 (43.33–139.80)	<0.001*
D-Dimer (*N* = 25) (μg/mL FEU)	4.47 ± 1.98 4 (3.07–5) N = (9)	3.05 ± 2.1 2.67 (1.7–4) N = (16)	0.0729
Wells' Score	4.82 ± 1.89 5 (3–6)	3.68 ± 1.67 4.5 (2–4.5)	0.014*

P-LCR = Platelet Large Cell Ratio, LPC = Large Platelet Count, PDW = Platelet Distribution Width, MPV = Mean Platelet Volume, PCT = Plateletcrit, TLC = Total Leukocyte Count, MLPR = Monocyte-to-Large Platelet Ratio, NLR = Neutrophil-to-Lymphocyte Ratio, PLR = Platelet-to-Lymphocyte Ratio, LPLR = Large Platelet-to-Lymphocyte Ratio, μg/mL FEU = micrograms per milliliter (fibrinogen equivalent units).

### 3.3 Diagnostic performance

Receiver operating characteristic (ROC) analysis demonstrated variable diagnostic performance across the evaluated hematological markers. Among individual parameters, MLPR showed the highest diagnostic accuracy (AUC: 0.8543), followed by monocyte count (AUC: 0.8207) and lymphocyte count (AUC: 0.7764), while total leukocyte count (TLC) showed moderate performance (AUC: 0.7314). In contrast, platelet-derived indices including PLR, LPLR, LPC, and PCT demonstrated poor or inverse discriminative ability, with AUC values below 0.40. Notably, MLPR outperformed conventional hematological parameters, supporting its potential diagnostic value. The combined lymphocyte–MLPR model demonstrated the highest performance (AUC: 0.9071) among the evaluated markers in this exploratory cohort. Using cutoffs of MLPR ≥0.734 and lymphocyte count ≥1.78 × 10^3^/μL, the combined model achieved 92% sensitivity and 85.7% specificity. The Wells score demonstrated moderate discriminatory performance (AUC: 0.6929, 95% CI: 0.548–0.837; *P* = 0.010), while the combination of Wells score and MLPR demonstrated higher discriminatory performance (AUC: 0.8729, 95% CI: 0.775–0.970; *P* < 0.001). The AUC of the combined Wells score–MLPR model was significantly higher than that of the Wells score alone (*P* = 0.0207). At the combined cutoff of MLPR ≥0.734 and Wells' score ≥ 5.0, the Wells score–MLPR model achieved 96.0% sensitivity, 75.0% specificity, 77.4% PPV, and 95.5% NPV. Overall, MLPR and its combinations with lymphocyte count or Wells score demonstrated promising diagnostic performance in this exploratory cohort compared with traditional leukocyte- and platelet-based parameters. ROC analysis of D-dimer, available for 25 patients, showed moderate diagnostic performance (AUC: 0.7222, 95% CI: 0.517–0.926, *P* = 0.0326) (Table 3, Table 4 and Fig. 2).

**Table 3:** ROC curve analysis of inflammatory markers for diagnosing PE.

Marker	AUC	SE	CI 95	P value
TLC (10^3^/μL)	0.7314	0.0728	0.588–0.874	0.0015
Monocyte (10^3^/μL)	0.8207	0.0596	0.703–0.937	<0.001
Neutrophils (10^3^/μL)	0.6743	0.0793	0.518–0.829	0.0280
Lymphocyte (10^3^/μL)	0.7764	0.0630	0.652–0.899	<0.001
LPC (10^3^/μL)	0.3300	0.0755	0.182–0.478	0.0243
PCT (%)	0.3607	0.0777	0.208–0.513	0.0730
MLPR	0.8543	0.0519	0.752–0.956	<0.001
PLR	0.2326	0.0677	0.099–0.365	<0.001
LPLR	0.1170	0.0453	0.028–0.205	<0.001
D-Dimer (*N* = 25)	0.7222	0.104	0.517–0.926	0.0326
Lymphocyte + MLPR	0.9071	0.0405	0.827–0.986	<0.001
Wells' Score	0.6929	0.0739	0.548–0.837	0.010
Wells' Score + MLPR	0.8729	0.0497	0.775–0.970	<0.001

TLC = Total Leukocyte Count, LPC = Large Platelet Count, PCT = Plateletcrit, MLPR = Monocyte-to-Large Platelet Ratio, PLR = Platelet-to-Lymphocyte Ratio, LPLR = Large Platelet-to-Lymphocyte Ratio.

**Table 4:** Diagnostic performance metrics of select markers for PE detection.

Marker	Cut off	Sensitivity % (95% CI)	Specificity % (95% CI)	PPV % (95% CI)	NPV % (95% CI)
TLC (10^3^/μL)	≥8.81	88.0 (68.8–97.5)	64.3 (44.1–81.4)	68.8 (50.0–83.9)	85.7 (63.7–97.0)
Monocyte (10^3^/μL)	≥0.52	80.0 (59.3–93.2)	71.4 (51.3–86.8)	71.4 (51.3–86.8)	80.0 (59.3–93.2)
Lymphocyte (10^3^/μL)	≥2.23	48.0 (27.8–68.7)	92.9 (76.5–99.1)	85.7 (57.2–98.2)	66.7 (49.8–80.9)
MLPR	≥0.734	96.0 (79.6–99.9)	64.3 (44.1–81.4)	70.6 (52.5–84.9)	94.7 (74.0–99.9)
D-Dimer (N = 25)	≥3	88.9 (51.8–99.7)	62.5 (35.4–84.8)	57.1 (28.9–82.3)	90.9 (58.7–99.8)
Lymphocyte + MLPR	MLPR ≥0.734 Lymph ≥1.78	92.0 (74.0–99.0)	85.7 (67.3–96.0)	85.2 (66.3–95.8)	92.3 (74.9–99.1)
Wells' Score + MLPR	MLPR ≥0.734 Wells ≥5.0	96.0 (79.6–99.9)	75.0 (55.1–89.3)	77.4 (58.9–90.4)	95.5(77.2–99.9)

TLC = Total Leukocyte Count, MLPR = Monocyte-to-Large Platelet Ratio.

**Fig. 2:**
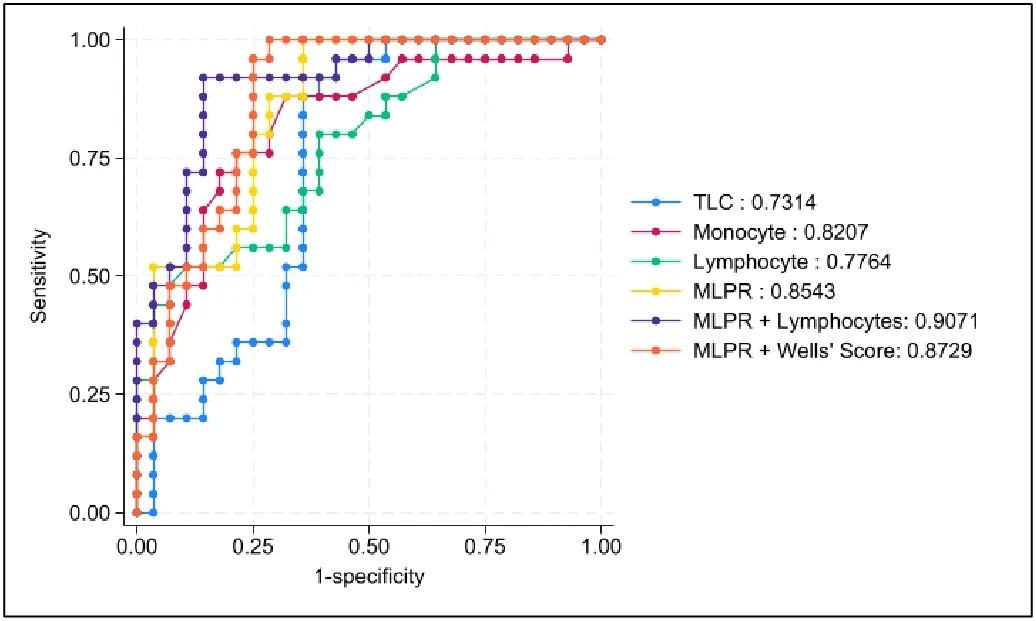
Receiver operating characteristic (ROC) curves of evaluated biomarkers for the diagnosis of pulmonary embolism.

### 3.4 Internal validation

Internal validation was performed using bootstrap resampling with 1000 iterations. For MLPR, bootstrap validation yielded an AUC of 0.854 (95% CI, 0.749–0.959), closely mirroring the original AUC of 0.854 (95% CI, 0.752–0.956). Similarly, the combined lymphocyte–MLPR model demonstrated a bootstrap-validated AUC of 0.907 (95% CI, 0.823–0.992), which was highly consistent with the original estimate of 0.907 (95% CI, 0.827–0.986). For the combined Wells score–MLPR model, bootstrap validation yielded an AUC of 0.873 (95% CI, 0.779–0.967), closely corresponding to the original AUC of 0.873 (95% CI, 0.776–0.970).At the predefined optimal cutoff (MLPR ≥0.734 and lymphocyte count ≥1.78 × 10^3^/μL), bootstrap-derived sensitivity and specificity were 92.0% (95% CI, 81.0–100.0%) and 85.7% (95% CI, 72.2–99.2%), respectively. The substantial overlap between the original and bootstrap-derived confidence intervals indicates stable model performance and supports the robustness of the findings despite the limited sample size.

### 3.5 Regression analysis

Predictor variables were selected for logistic regression analysis based on their diagnostic accuracy, as determined by the Area Under the Receiver Operating Characteristic Curve (AUC) from preliminary ROC analyses. Variables with higher discriminatory performance were then evaluated using univariable logistic regression to assess their individual associations with the outcome. Accordingly, total leukocyte count (TLC), monocyte count, lymphocyte count, monocyte-to-large platelet ratio (MLPR), and a combined lymphocyte–MLPR metric were analyzed. Given the relatively small sample size, regression coefficients were reported instead of odds ratios to avoid potential overestimation and instability of effect size estimates. All variables demonstrated statistically significant associations with the outcome, with MLPR and the combined lymphocyte–MLPR index showing the strongest overall predictive performance. Monocyte count also showed a strong association, while lymphocyte count and TLC demonstrated weaker but still significant relationships. Overall, the combined lymphocyte–MLPR and Wells'-MLPR metrics provided the greatest explanatory performance among the evaluated predictors (Table 5).

**Table 5:** Univariable logistic regression predicting pulmonary embolism (PE).

Predictor	Coefficient	Std. Error	*P*-value	95% CI	Pseudo R^2^
TLC (10^3^/μL)	0.2767	0.0983	0.005	0.084–0.469	0.1300
Monocytes (10^3^/μL)	4.7718	1.3881	0.001	2.051–7.492	0.2344
Lymphocytes (10^3^/μL)	1.4227	0.4573	0.002	0.526–2.319	0.1937
MLPR	3.8232	1.1276	0.001	1.613–6.033	0.3040
Lymphocyte + MLPR	5.3822	1.2680	<0.001	2.896–7.867	0.4021
Wells' Score	0.370	0.1709	0.030	0.035–0.705	0.0728
Wells' Score + MLPR	6.053	1.6041	<0.001	2.909–9.197	0.3547

TLC = Total Leukocyte Count, MLPR = Monocyte-to-Large Platelet Ratio.

Multivariable logistic regression was performed to assess the association between MLPR and pulmonary embolism after adjustment for Wells score, age, and gender. In the adjusted model, MLPR remained significantly associated with PE (coefficient = 3.834, 95% CI: 1.466–6.201; *P* = 0.002), whereas Wells score (coefficient = 0.281, 95% CI: −0.122–0.684; *P* = 0.172), age (coefficient = −0.009, 95% CI: −0.049–0.031; *P* = 0.662), and gender (coefficient = −0.033, 95% CI: −1.614–1.549; *P* = 0.968) were not independently associated with PE. The overall model was statistically significant (LR χ^2^ = 24.77, *P* < 0.001; pseudo-R^2^ = 0.3379). Multicollinearity among the variables included in the multivariable model was assessed using variance inflation factors (VIFs) and tolerance values. VIFs were 1.16 for MLPR, 1.17 for Wells score, 1.07 for age, and 1.30 for gender, with corresponding tolerance values of 0.862, 0.858, 0.931, and 0.771, respectively. The mean VIF was 1.17, indicating no evidence of problematic multicollinearity.

## 4 Discussion

Pulmonary embolism (PE) presents a significant diagnostic challenge, especially when computed tomography pulmonary angiography (CTPA) is unavailable or contraindicated due to factors such as renal impairment, contrast allergies, or pregnancy. In such situations, clinicians often rely on clinical assessments, D-dimer testing, and alternative imaging modalities, which may lack the specificity and sensitivity of CTPA. The monocyte-to-large-platelet ratio (MLPR) has emerged as a potential biomarker for PE diagnosis, as demonstrated by multiple studies, including our own. This exploratory study compares our findings with previous research to assess the consistency and variability of MLPR's diagnostic value in a broader suspected PE population, not specific to patients with acute exacerbation of chronic obstructive pulmonary disease (AECOPD).

Our study reviewed the records of 115 patients, of whom 53 met the inclusion criteria. Demographic factors such as age (47.4 ± 16.8 vs. 51.5 ± 18.6 years, *P* = 0.345) and male gender distribution (52% vs. 28.5%, *P* = 0.099) did not significantly differ between the two groups. Among the hematological indices evaluated, the monocyte-to-large platelet ratio (MLPR) was significantly elevated in PE patients (1.19 ± 0.45 vs. 0.61 ± 0.39, *P* < 0.001), as were total leukocyte, monocyte, neutrophil, and lymphocyte counts, suggesting a pro-inflammatory and pro-thrombotic profile. In contrast, platelet-to-lymphocyte ratio (PLR), large platelet-to-lymphocyte ratio (LPLR), and large platelet count (LPC) were significantly lower in PE patients. Notably, the neutrophil-to-lymphocyte ratio (NLR) did not differ significantly between groups (*P* = 0.4235).

Receiver operating characteristic (ROC) analysis showed that MLPR had the highest diagnostic accuracy among individual hematological markers (AUC: 0.8543, 95% CI: 0.752–0.956, *P* < 0.001), followed by monocyte count (AUC: 0.8207) and lymphocyte count (AUC: 0.7764). Moreover, combining MLPR with lymphocyte count further improved diagnostic performance, yielding an AUC of 0.9071 (95% CI: 0.827–0.986, *P* < 0.001), with 92% sensitivity and 85.7% specificity using cutoffs of MLPR ≥0.734 and lymphocyte count ≥1.78 × 10^3^/μL. In contrast, platelet-derived indices such as PLR and LPLR demonstrated poor or inverse discriminative performance.

To assess whether MLPR may provide additional diagnostic information beyond clinical pretest probability, the Wells score was evaluated alone and in combination with MLPR. The Wells score demonstrated moderate discriminatory performance (AUC: 0.693, 95% CI: 0.548–0.837), whereas the combination of Wells score and MLPR achieved an AUC of 0.873 (95% CI: 0.775–0.970). The addition of MLPR significantly improved discrimination compared with the Wells score alone (*P* = 0.0207), suggesting potential incremental diagnostic value. D-dimer demonstrated an AUC of 0.722 (95% CI: 0.517–0.926) among the 25 patients for whom it was available. However, because D-dimer testing was not performed systematically in all participants, these data do not allow a reliable direct comparison between MLPR and D-dimer or conclusions regarding superiority. These findings suggest that MLPR may provide useful diagnostic information in patients with suspected PE, including information beyond clinical pretest probability as assessed by the Wells score. However, the results should be interpreted as exploratory given the small retrospective cohort and the limited availability of D-dimer measurements.

Our findings are consistent with those of Bialas et al., who also investigated MLPR in PE but specifically in patients with AECOPD. Their study reported an exceptionally high AUC of 0.945, with 100% sensitivity and 85.7% specificity, suggesting that MLPR may be particularly predictive in this subgroup. In contrast, our results indicate that while MLPR remains a valuable diagnostic marker in a general patient population, its sensitivity and specificity are somewhat lower than in AECOPD-associated PE. This discrepancy could be attributed to the heightened inflammatory and prothrombotic state in AECOPD, where monocyte activation and platelet turnover are more pronounced [20].

Similarly, Liu and Cheng examined MLPR in AECOPD patients with PE, reporting that MLPR was significantly elevated in PE cases, with an AUC of 0.855. At a cutoff value of >1.04%, MLPR demonstrated a sensitivity of 94.8% and specificity of 75.7%, further supporting its role as a diagnostic marker [18].

Further supporting MLPR, Zhang and Yang assessed this marker alongside neutrophil-to-lymphocyte ratio (NLR) and red cell distribution width (RDW) in elderly AECOPD patients with PE. They found higher MLPR, NLR, and RDW levels in patients with emboli from thrombus dislodgment versus in-situ clot formation, suggesting that these markers reflect a more pronounced systemic thromboinflammatory response associated with thrombus formation in the peripheral circulation and subsequent embolization, rather than localized in-situ pulmonary thrombosis. Although their group definitions differ from standard PE versus non-PE classifications, the study shows that a combined marker approach can improve diagnostic performance. In our study, we similarly combined MLPR with other inflammatory markers to compare confirmed PE and non-PE patients, demonstrating enhanced diagnostic accuracy [17].

All studies, including ours, confirm MLPR as a valuable biomarker for PE diagnosis. Studies on AECOPD-related PE (Liu & Cheng, Zhang & Yang, Bialas) reported higher diagnostic performance, likely due to heightened inflammation in COPD where monocyte activation and platelet turnover are more pronounced [17], [18], [20]. Our study expands MLPR's applicability beyond AECOPD, reinforcing its potential as a general diagnostic marker for PE.

### 4.1 Neutrophil to lymphocyte ratio

While our results highlight the potential role of the monocyte-to-large platelet ratio (MLPR) in pulmonary embolism, the neutrophil-to-lymphocyte ratio (NLR) did not differ significantly between PE and non-PE groups in our cohort. This observation is consistent with Signorelli et al., who reported no significant NLR difference between PE patients and hospitalized controls, likely reflecting comparable stress-related inflammatory responses in both groups [23]. Celik et al. found significant NLR differences in univariate analysis comparing confirmed and suspected PE cases, although this association was not sustained in multivariate models [24]. In contrast, studies comparing PE patients to healthy controls (Cavuş et al., Bakırci et al. and Shan et al.) observed elevated NLR levels in PE, which may be influenced by the absence of acute illness or stress in control subjects [25], [26], [27]. These findings emphasize the critical role of control group selection in interpreting NLR as a biomarker for PE.

### 4.2 Absolute lymphocyte count

Unlike NLR, Lymphocyte counts were found to be significantly higher among patients diagnosed with pulmonary embolism (PE) compared to those without, despite both groups exhibiting values within normal physiological limits. This elevation reached statistical significance and was further supported by receiver operating characteristic (ROC) analysis, which indicated moderate ability to distinguish between PE and non-PE cases. In addition, logistic regression, both univariable and multivariable models identified lymphocyte count as an independent factor associated with PE, suggesting that even subtle shifts within the normal range may carry diagnostic value.

Although this contrasts with several previous reports that linked pulmonary embolism to lymphopenia, it is important to note that lymphopenia has been predominantly reported in studies assessing mortality outcomes or poor adverse events in patients with confirmed PE, rather than in diagnostic studies comparing PE and non-PE populations [28], [29], [30]. This distinction suggests that reduced lymphocyte counts may be more closely associated with disease severity and prognosis, rather than serving as a reliable marker for initial diagnosis.

However, among diagnostic studies, this discrepancy may be attributed to differences in study populations and objectives. Four prior studies examined lymphocyte counts in relation to pulmonary embolism (PE) by comparing patients with and without the diagnosis. Two of these, conducted among individuals already hospitalized for cardiology or neurology conditions, reported no significant differences in lymphocyte levels between groups [31], [32]. A third study, limited to trauma patients, observed lower lymphocyte counts in those with PE [33]. All three focused on high-risk, hospitalized populations and were primarily designed to develop prediction models rather than to assess diagnostic markers of acute PE.

In contrast, one study aimed to evaluate hemogram parameters specifically for diagnosing PE in patients presenting suspected [19]. While it reported lower lymphocyte counts in PE patients, it notably excluded individuals with conditions such as bone fractures which is already a risk factor for VTE. Additionally, the PE group in that study was significantly older than the non-PE group, which may have contributed to the observed lymphopenia.

### 4.3 Lymphocytes dynamics

An additional finding of the present study was that combining lymphocyte count with MLPR improved the diagnostic performance of MLPR, particularly its specificity. This finding was associated with a higher lymphocyte count among patients with PE, which contrasts with the lymphopenia commonly reported in previous studies. This apparent lymphocytosis may be related to the dynamic nature of the lymphocyte response during acute stress and the timing of blood sampling relative to symptom onset.

Acute physiological or psychological stress can transiently increase circulating lymphocyte counts through catecholamine-mediated redistribution during the early stress response [34], [35], [36], [37], [38]. This initial lymphocytosis may subsequently be followed by a decline in lymphocyte counts as cortisol-mediated effects become more prominent, potentially resulting in lymphopenia [28], [39], [40]. Therefore, the timing of blood sampling relative to symptom onset may partly explain the discrepancy between our findings and previous reports [41]. However, because the retrospective design did not allow reliable assessment of the interval between symptom onset and blood sampling, this explanation remains hypothetical.

### 4.4 Potential mechanisms linking MLPR and PE

The association between MLPR and PE may reflect the interplay between inflammation and thrombosis. Monocytes contribute to inflammatory and procoagulant pathways, including tissue factor expression and release of proinflammatory mediators. Increased monocyte counts have also been reported in patients with VTE and PE [20], [42], [43], [44], [45], [46], [47], [48]. Moreover, Granger et al. reported that highly purified human blood monocytes are capable of extracellular trap release in response to several stimuli, and that these monocyte extracellular traps demonstrate procoagulant activity, which broadens the range of the thrombogenic properties of monocytes [49].

Białas et al. observed a higher absolute monocyte count in patients with PE [20]. These observations, as well as evidence concerning the role of monocytes and platelet–monocyte crosstalk in thrombus formation, constitute the rationale for developing the MLPR and assessing its usefulness for diagnostic purposes [49], [50], [51]. Furthermore, monocyte count has been investigated in studies to assess the severity of PE and it has been emphasized that it may have some predictive value for VTE [52].

On the other hand, large platelets, which are newly formed and more thrombogenic, may contribute to this association [53], [54], [55]. They exhibit greater expression of platelet activation markers and mediators that promote platelet aggregation and thrombosis [56], [57]. Large platelets may also be preferentially consumed during thrombus formation, resulting in a reduced circulating large-platelet fraction, consistent with evidence that larger platelets undergo more rapid and complete aggregation [21], [22].

Additionally, the findings of Karakurt et al. support the significance of monocytes and platelet large cell ratio (P-LCR) in PE pathophysiology. Elevated monocyte levels indicate increased inflammatory activity, while the reduction in large platelets suggests enhanced platelet consumption, a hallmark of ongoing thrombogenesis. Their study reinforces the role of monocyte-platelet interactions in the disease process, highlighting MLPR as a reflection of both inflammatory and thrombotic pathways [19].

### 4.5 Limitations

Our study had several limitations. First, it was retrospective and conducted at a single center, which may limit generalizability. Of the 115 initially reviewed records, 53 patients (25 with PE and 28 without PE) constituted the final analyzable cohort. The availability of the platelet parameters required to calculate MLPR also depended on laboratory analyzer-specific data, which may have contributed to the exclusion of otherwise potentially eligible patients. Additionally, a comprehensive comparison between MLPR and D-dimer performance was not feasible, as D-dimer testing was available for only 25 patients. This limitation arose because, in clinical practice, D-dimer testing is typically reserved for patients with a low clinical probability of PE, as determined by the Wells criteria. Consequently, we were also unable to reliably assess combined models incorporating D-dimer. Furthermore, the relatively small sample size increases the risk of model overfitting and may limit the stability of regression estimates and diagnostic cutoff values. Although the multivariable analysis incorporated Wells score, age, and gender, other potential clinical confounders, including previous venous thromboembolism and comorbidities, could not be comprehensively incorporated because of the limited sample size and retrospective availability of clinical data; therefore, residual confounding cannot be excluded. Accordingly, this study does not allow conclusions regarding the superiority of MLPR over D-dimer. External validation was not performed, and therefore the reported diagnostic performance metrics should be considered exploratory and interpreted cautiously until confirmed in larger independent cohorts. Nonetheless, strengths of this study include the evaluation of multiple hematologic indices, many of which remain underreported in the literature, and the investigation of these markers in a general population rather than one limited to patients with AECOPD. These findings support the need for larger prospective multicenter studies to validate and expand upon our observations.

## 5 Conclusion

In conclusion, our findings suggest that the combination of MLPR and lymphocyte count significantly improved diagnostic performance for PE, with an AUC of 0.90, sensitivity of 92%, and specificity of 85.7%. This composite approach showed encouraging diagnostic performance in this cohort, although these findings should be considered exploratory given the relatively small sample size and retrospective study design. In addition, the combination of MLPR and the Wells score improved diagnostic discrimination compared with the Wells score alone, with an AUC of 0.87. Given their accessibility and low cost, MLPR and lymphocyte count may represent accessible adjunctive biomarkers that warrant further investigation alongside established diagnostic strategies. Further prospective multicenter studies are required to validate these findings and clarify the potential diagnostic and prognostic roles of MLPR in PE. MLPR should not be considered a replacement for established diagnostic algorithms or imaging modalities, but rather a potentially supportive biomarker that requires further validation.

MLPR should not be considered a replacement for established diagnostic algorithms or imaging modalities, but rather a potentially supportive biomarker that requires further validation.

## CRediT authorship contribution statement

**Mohamed K.A. Awad:** Writing – original draft, Methodology, Formal analysis, Data curation, Conceptualization. **Reem H. El Kabarity:** Writing – review & editing, Supervision, Project administration, Investigation, Conceptualization. **Hadeel Magdy Abdelhamid:** Writing – review & editing, Supervision, Methodology, Investigation. **Azza M. Youssef:** Writing – review & editing, Software, Methodology, Investigation.

## Consent for publication

Not applicable. This study used anonymized retrospective data, and no individual patient data is presented.

## Ethical approval

This study was approved by the Research Ethics Committee (REC), Faculty of Medicine, Ain Shams University and was registered on ClinicalTrials.gov (Identifier: NCT07023705). Due to the retrospective nature of the study, the requirement for informed consent was waived. All methods were carried out in accordance with relevant guidelines and regulations, including the Declaration of Helsinki.

## Ethical statement

This study was approved by the Research Ethics Committee (REC) FWA00017585, Faculty of Medicine, Ain Shams University, and was registered on ClinicalTrials.gov (Identifier: NCT07023705). Due to the retrospective nature of the study, the requirement for informed consent was waived. Patient medical records were reviewed to identify eligible cases. All methods were carried out in accordance with relevant guidelines and regulations, including the Declaration of Helsinki.

## Funding sources

The authors reported no financial support.

## Declaration of competing interest

The authors have no conflicts of interest to disclose.

## Data Availability

The datasets generated and/or analyzed during the current study are not publicly available due to patient confidentiality and institutional ethical restrictions but are available from the corresponding author on reasonable request.
